# Time, Ideologies, and Care: Gendered Patterns of Parental Involvement in the UK and Portugal

**DOI:** 10.3390/bs15091204

**Published:** 2025-09-04

**Authors:** Mariana Pinho, Inês Lourenço, Marisa Lousada

**Affiliations:** 1 ECOMARE, Centre for Environmental and Marine Studies, Department of Biology, University of Aveiro, 3810-193 Aveiro, Portugal; 2 School of Health Sciences (ESSUA), University of Aveiro, 3810-193 Aveiro, Portugal; ineslourenco@ua.pt; 3 RISE-Health, School of Health Sciences (ESSUA), University of Aveiro, 3810-193 Aveiro, Portugal; marisalousada@ua.pt

**Keywords:** caregiving, parenting, gender ideologies, essentialism: childcare, UK, Portugal

## Abstract

This study examines cross-national differences in parental involvement, work hours, and gender ideologies among parents in the UK and Portugal. Findings revealed that UK parents, particularly mothers, reported higher childcare involvement, while Portuguese parents worked more paid hours, reflecting fewer structural childcare constraints. Despite mothers in both countries endorsing more egalitarian gender ideologies than fathers, Portuguese parents overall held more egalitarian views and lower essentialist beliefs. Surprisingly, British fathers reported greater involvement in physical childcare than their Portuguese counterparts. Gender ideologies negatively predicted partner childcare hours, indicating compensatory dynamics, with significant mediation by work hours only in the UK. This suggests that egalitarian gender beliefs alone might be insufficient for achieving equality in family roles without corresponding sociopolitical frameworks to enable equitable practices. These results highlight the influence of national context and gender beliefs on family labour divisions and underscore the need for policies that support more equitable sharing of paid and unpaid responsibilities across both countries.

## 1. Introduction

Over recent decades, the gender gap in both paid and family work has significantly narrowed, driven by rising female labour force participation and increased paternal involvement in caregiving (Allen & Stevenson, 2023; Schoppe-Sullivan & Fagan, 2020). This convergence reflects a broader cultural shift toward the belief that breadwinning and caregiving should be shared equally between mothers and fathers (Harrington, 2022; Knight & Brinton, 2017). Nevertheless, traditional gender roles remain deeply embedded in family life, with mothers still performing the bulk of housework and childcare (Negraia et al., 2018; Yavorsky et al., 2015). This persistent inequality disadvantages mothers professionally and limits fathers’ opportunities to engage deeply in caregiving (Deutsch & Gaunt, 2020). Much existing research has focused on economic and structural factors for these enduring disparities (e.g., Hook, 2006; Sullivan et al., 2018), leaving social psychological mechanisms less explored.

Furthermore, there is limited cross-national comparative understanding of how social psychological mechanisms, such as gender ideologies, shape the division of paid work and childcare. Specifically, much of the existing literature focuses on structural and policy-level explanations in Anglo-Saxon contexts like the UK (e.g., Bendall & Mitchell, 2023; Hook, 2006), remaining an insufficient exploration of how these internalised beliefs operate within different cultural and policy environments, such as in Southern European countries like Portugal. The current comparative study explores how gender ideologies and biological essentialism differ within two national contexts and how they relate to caregiving behaviour, contributing to the understanding of social psychological mechanisms behind persistent gender inequalities in family life.

### 1.1. Gender Ideologies, Biological Essentialism, and the Division of Paid Work and Childcare

Parental involvement in childcare is strongly influenced by structural factors, such as the number of hours spent in paid employment, with increased work hours often corresponding to reduced time available for caregiving (Bünning, 2020; Gaunt & Deutsch, 2024; Kaplan & Offer, 2022). However, time availability alone does not fully account for the persistent gendered patterns observed in caregiving responsibilities. Emerging research suggests that broader socio-cultural factors, such as value systems, identities, and ideological beliefs, also play a critical role in shaping how parents navigate work and care commitments (Gaunt & Scott, 2014; Huffman et al., 2014). Contemporary scholarship on gender ideologies highlights the socially constructed nature of gender roles and their influence on the labour division within households. Gender ideologies reflect individuals’ beliefs about the appropriate roles and responsibilities of men and women and continue to shape expectations around paid work and caregiving, with fathers as breadwinners and mothers as primary caregivers (Davis & Greenstein, 2009). While egalitarian ideologies have become more prevalent, with studies linking egalitarian ideologies to more equal parenting involvement (Bornatici & Zinn, 2025; Wang & Cheung, 2023), traditional views persist (particularly concerning motherhood) (e.g., Cunningham, 2008).

Biological essentialism reinforces these patterns by attributing gendered behaviours and capabilities to innate biological differences between men and women, with mothers assuming more caregiving tasks and reducing work hours, while fathers engage less in childcare (Deutsch, 1999; Joecks et al., 2024; Thorsteinsen et al., 2022). Scholars argue that these beliefs legitimise gender inequality by naturalising social constructs, thereby discouraging institutional and cultural changes that might promote more equitable divisions of labour (Gaunt & Deutsch, 2024; Risman et al., 2018). The persistence of biological essentialism contributes to a cultural climate in which deviations from traditional gender roles, such as role-reversed couples, are still a minority and often stigmatised (Rochlen et al., 2010). Consequently, addressing the unequal division of labour requires a critical interrogation of the gender ideologies and essentialist beliefs that underpin social expectations and institutional practices. Our study was designed to explore how gender ideologies differ within two national contexts (the UK and Portugal) and how they relate to caregiving behaviour, contributing to the understanding of social psychological mechanisms behind persistent gender inequalities in family life.

### 1.2. Navigating Paid Work and Childcare in the UK and Portugal

Childcare systems in the United Kingdom and Portugal differ in accessibility, affordability, and their underlying sociopolitical ideologies, shaping divergent gender role expectations and labour market outcomes. In the UK, the high cost of childcare significantly limits maternal full-time employment (Costa Dias et al., 2020; Eurofound, 2021). This economic constraint, coupled with persistent cultural ambivalence toward full-time maternal employment, contributes to elevated rates of part-time work among women (Coleman et al., 2022; Phillips et al., 2018). These trends reflect a broader cultural model where caregiving is seen as a maternal domain, reinforced by labour market structures that lack adequate flexibility and institutional support for caregiving responsibilities (Chung et al., 2021). Although gender equality initiatives have increased women’s representation in leadership, particularly in corporate sectors, women remain underrepresented in top executive roles, and the gender pay gap persists (Office for National Statistics, 2024). These structural and cultural factors combine to sustain traditional gender role schemas, limiting shifts toward egalitarian paid work participation. Conversely, Portugal’s more collectivist orientation and strong public investment in early childhood education have facilitated a dual-earner family model (European Institute for Gender Equality [EIGE], 2024). Universal access to free pre-primary education for children aged 3 to 5, alongside the progressive expansion of free childcare for those under 3, has significantly reduced the work–family conflict for mothers (European Commission [EC], 2024). Early childhood professionals are highly educated, and the national curriculum promotes inclusive developmental goals, reflecting a social policy framework that supports gender equality through institutional design (Lopes da Silva et al., 2016; Organisation for Economic Co-operation and Development [OECD], 2012). While women in Portugal face a persistent gender pay gap and remain underrepresented in leadership positions, their labour market participation is less constrained by childcare barriers compared to the UK (Eurofound, 2024). Nonetheless, cultural expectations around maternal caregiving endure, and women continue to shoulder a disproportionate share of unpaid domestic labour (Cunha & Atalaia, 2019; Pinho et al., 2025). Taken together, these cross-national contrasts highlight the culturally embedded nature of gender role beliefs and their interaction with policy infrastructure. While both countries have made legislative progress, the UK’s liberal market model continues to rely on private solutions that reinforce “traditional” gender division of family roles, whereas Portugal’s public welfare orientation supports more equitable participation, albeit within enduring normative frameworks.

## 2. Method

### 2.1. Participants and Procedure

The criteria for inclusion in this study in both countries were those who were married or cohabiting with their different-gender partner and had at least one biological child aged 4 years or younger. Participants were asked to complete a questionnaire on the ways that families organise work and childcare. In the UK, they were mostly completed online, while in Portugal, they were primarily completed using a paper version.

For the UK sample, recruitment was performed through emails sent to the 800,000 nationally representative members of the YouGov UK panel. Data from 1049 parents (557 men and 492 women) were collected, and families had between 1 and 8 children (*M* = 1.96, *SD* = 0.88). More than half of the parents worked full-time (58.9%), earned between GBP 1001 and GBP 2600 (52%), and held a university degree (65%). On average, parents worked 30 h per week.

Recruitment in Portugal was carried out through advertisements in childcare centres and preschools. Data were collected from 155 parents (80 women and 75 men) for the Portuguese sample. Most parents residing in Portugal had a university degree (62.1%), worked full-time (92.2%), and earned a monthly individual income between GBP 891 to GBP 2083 (58%). The number of children in the family ranged from 1 to 4 (*M* = 1.72, *SD* = 0.69). Participants’ average weekly working hours were 36.

Socio-demographic characteristics of both samples can be found in Table 1. Participation in this study was voluntary and anonymous, and lasted an average of 15 min.

### 2.2. Measures

*Time Investment in Work and Childcare.* Participants reported the number of hours they and their partners worked for pay each week. They also indicated how many hours per week they spent alone with their child while the child was awake, as well as the number of hours their partner spent alone with the child.

*Division of Childcare and Housework Tasks.* The division of family labour was measured using the “Who-does-what?” task performance (Gaunt & Pinho, 2018), which includes 24 tasks. Participants were asked: “*In the division of labour between you and your partner, which of you does each of these tasks?*”. Responses were given on a 5-point scale ranging from 1 = *Almost always my partner*, through 3 = *Both of us equally*, to 5 = *Almost always myself*. Participants could also select 9 if a task was not relevant to their child; these responses were treated as missing data. The scale comprises four sub-dimensions: housework (e.g., cooking and cleaning), physical care (e.g., feeding and bathing), emotional care (e.g., playing and helping with social/emotional problems), and responsibility (e.g., planning activities and choosing daycare). Mean scores were calculated for each sub-dimension to assess participants’ involvement. Cronbach’s alpha for the four subscales was 0.76, 0.88, 0.79, and 0.87, respectively. Additionally, an overall childcare involvement was computed by averaging all relevant childcare items, with a Cronbach’s alpha of 0.94.

*Egalitarian Gender Ideologies.* Participants’ beliefs about gender roles were assessed using a five-item scale adapted from Gaunt (2006), which captures both traditional and egalitarian perspectives (e.g., “*Men and women should share housework when both are employed*”; “*Marriage is a partnership in which spouses should share the economic responsibility for supporting the family*”). Responses were rated on a 5-point scale from 1 = *Strongly Disagree* to 5 = *Strongly Agree*. Items were recoded so that higher scores indicated more egalitarian gender attitudes. The average of four retained items was used to calculate each participant’s gender ideology score. Cronbach’s alpha for this measure was 0.72.

*Biological Essentialism.* Parents’ beliefs about inherent differences between men and women in their parenting abilities were measured using a seven-item scale developed by Gaunt (2006). The items reflect essentialist views of caregiving (e.g., “*Mothers are instinctively better caretakers than fathers*”; “*Fathers have to learn what mothers are able to do naturally in terms of childcare*”). Responses were given on a 5-point scale from 1 *= Strongly Disagree* to 5 = *Strongly Agree* and recoded so that a higher score reflected stronger biological essentialist beliefs. An average score across the seven items was calculated to represent each participant’s level of biological essentialism. Cronbach’s alpha for this measure was 0.86.

*Socio-demographic Variables.* Participants indicated their age, occupation, and level of education. Participants also reported the gender and age of their child, the total number of children in the household, and their individual monthly income on a seven-point scale ranging from 1 (*less than EUR 590*) to 7 (*more than EUR 6720*).

## 3. Results

**Figure 1:**
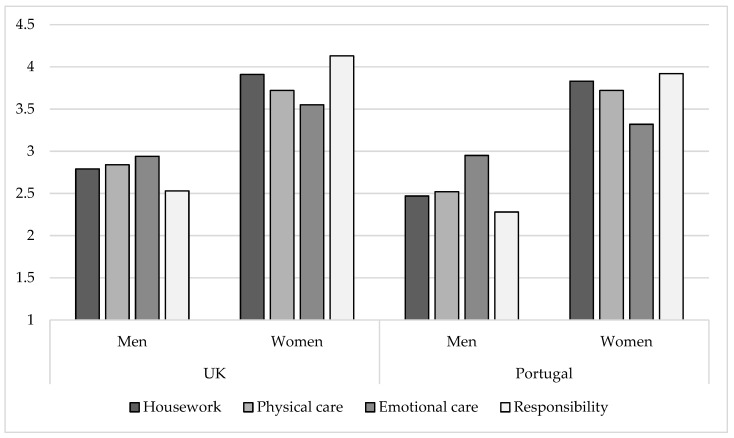
Housework and Childcare by Gender and Country. Note: Share of housework and childcare was measured on a scale from 1 = *Almost always my partner* to 5 = *Almost always myself*.

To analyse gender and family arrangement effects on couples’ share of housework and childcare, a set of 2 (Gender: Men vs. Women) × 2 (Country: UK vs. Portugal) between-participants analyses of variance (ANOVAs) was conducted (Figure 1).

Significant main effects and interactions were followed up with simple-effects analysis. Bootstrap resampling was used to mitigate the effects of unequal sample sizes, enabling non-parametric inference by generating empirical sampling distributions that do not rely on assumptions of homoscedasticity or normality. The findings showed a significant main effect of gender (*F* (1, 1177) = 25.07, *p* < 001) and country (*F* (1, 1177) = 16.50, *p* < 0.001). Post hoc Tukey tests indicated that parents in the UK spent significantly more weekly hours looking after their children (*M* = 21.74) than parents in Portugal (*M* = 15.59), and mothers in both countries (*M* = 26.24) spent more hours on childcare than fathers (*M* = 16.19). This effect was qualified by a significant gender x country interaction (*F* (1, 1177) = 3.307, *p* = 0.08), suggesting that mothers in the UK (*M* = 27.51) spent more hours on childcare than mothers in Portugal (*M* = 18.14, SD = 17.44) (*t* (1, 1177) = 17.19, *p* < 001) and fathers in the UK (*M* = 16.62) (*t* (742) = −9.87, *p* < 0.001).

When analysing weekly working hours, a main effect of gender (*F* (1, 1189) = 24.82, *p* < 0.001) and country were found (*F* (1, 1189) = 21.73, *p* < 0.001). Post hoc Tukey tests showed that fathers in the UK (*M* = 36) worked more hours a week than mothers in the UK (*M* = 24.33), while no gender differences were found among parents in Portugal. Additionally, parents in Portugal worked more weekly hours (*M* = 36.66) than parents in the UK (*M* = 30.51). This effect was qualified by a significant gender x country interaction (*F* (1, 1189) = 11.12, *p* < 0.001), indicating that mothers in Portugal (*M* = 35.55) worked significantly more hours than mothers in the UK (*M* = 24.33) (*t* (185) = −8.38, *p* < 0.001).

The analysis also revealed a main effect of gender (*F* (1, 1197) = 330.66, *p* < 0.001) and country on involvement in housework (*F* (1, 1197) = 8.56, *p* = 0.003) (Figure 1). Post hoc Tukey tests indicated that in the UK (*M* = 3.31), parents were significantly more involved in housework than parents in Portugal (*M* = 3.17). Additionally, in both countries, mothers did significantly more housework (*M* = 3.90) than fathers (*M* = 2.75). This effect was qualified by a significant gender x country interaction, *F* (1, 1197) = 10.64*, p =* 0.001, suggesting that fathers in the UK performed significantly more housework (*M* = 2.75) than fathers in Portugal (*M* = 32.47), *t* (628) = 3.55, *p* < 0.001), while no significant differences were found for mothers’ housework involvement in both countries.

Similar results were found for involvement in childcare tasks (Figure 1). A main effect of gender was found in physical (*F* (1, 1197) = 275.63, *p* < 001), emotional (*F* (1, 1197) = 110.30, *p* < 0.001), and responsibility-related childcare tasks (*F* (1, 1197) = 772.23*, p* < 0.001). Post hoc Tukey tests indicated that in both countries, mothers were significantly more involved in physical, emotional, and responsibility than fathers. A main effect of country was also found for the three types of childcare tasks analysed (*F* (1, 1197) = 6.43, *p* = 0.011; *F* (1, 1197) = 5.36, *p* = 0.021; *F* (1, 1197) = 15.40, *p* < 0.001; for physical, emotional and responsibility, respectively), suggesting that parents in the UK (*M* = 3.25; *M* = 3.22; *M* = 3.28) were more involved in physical, emotional and responsibility-related tasks than parents in Portugal (*M* = 3.14; *M* = 3.14; *M* = 3.12, respectively).

These effects were qualified by significant gender x country interactions (*F* (1, 1197) = 6.743, *p* = 0.01; *F* (1, 1197) = 6.42, *p* = 0.011; for physical and emotional care, respectively). This meant that fathers in the UK performed significantly more physical care (*M* = 2.84) than fathers in Portugal (*M* = 2.52; *t* (628) = 3.82, *p* < 0.001), while mothers in the UK (*M* = 3.54) were significantly more involved in emotional childcare tasks than mothers in Portugal (*M* = 3.32; *t* (124) = 3.58, *p* < 0.001).

**Figure 2:**
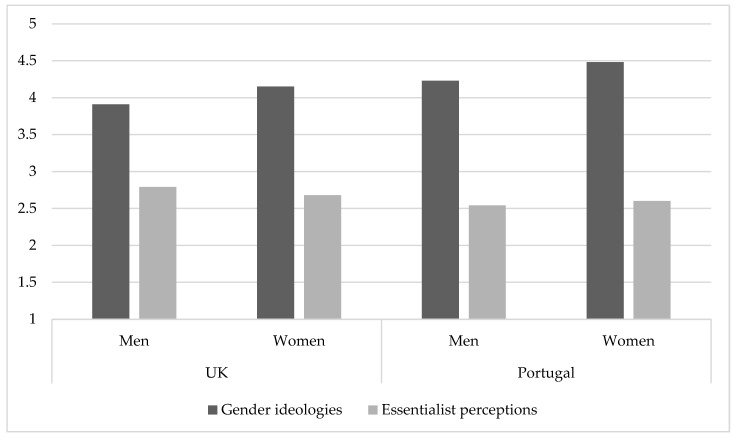
Egalitarian Gender Ideologies and Essentialist Perceptions by Gender and Country.

An additional analysis was conducted to explore gender and country effects on parents’ egalitarian gender ideologies and biological essentialism, through a set of two 2 (Gender: Men vs. Women) × 2 (Country: UK vs. Portugal) between-participants analyses of variance (ANOVAs) (Figure 2).

Significant main effects and interactions were followed up with simple-effects analysis. The results revealed a main effect of gender (*F* (1, 1180) = 13.62, *p* < 0.001) for egalitarian gender ideologies and country for both (*F* (1, 1180) = 22.591, *p* < 0.001; *F* (1, 1177) = 5.95, *p* = 0.015; for egalitarian gender ideologies and essentialist perceptions, respectively). Mothers in both countries (*M* = 4.21) expressed higher egalitarian gender ideologies than fathers (*M* = 3.95). These results also indicated that Portuguese parents expressed higher levels of egalitarian gender ideologies (*M* = 4.36) and lower essentialist perceptions (*M* = 2.57) than parents in the UK (*M* = 4.03; *M* = 2.74, respectively). Regarding essentialist perceptions, this effect was driven by a significant difference among fathers, meaning that fathers in Portugal (*M* = 2.54) expressed significantly lower essentialist perceptions than fathers in the UK (*M* = 2.79) (*t* (112) = 3.10, *p* = 0.002).

**Figure 3:**
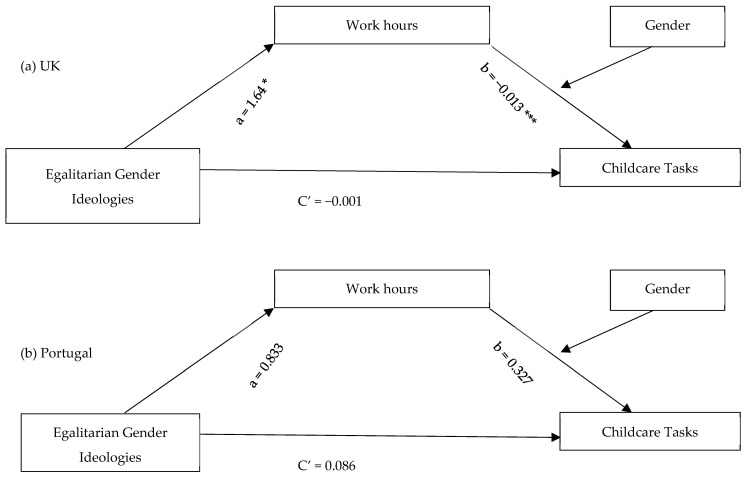
Conditional Indirect Effects of Egalitarian Gender Ideologies on Involvement in Childcare Tasks Through Work Hours. * *p* < 0.05; *** *p* < 0.001.

**Figure 4:**
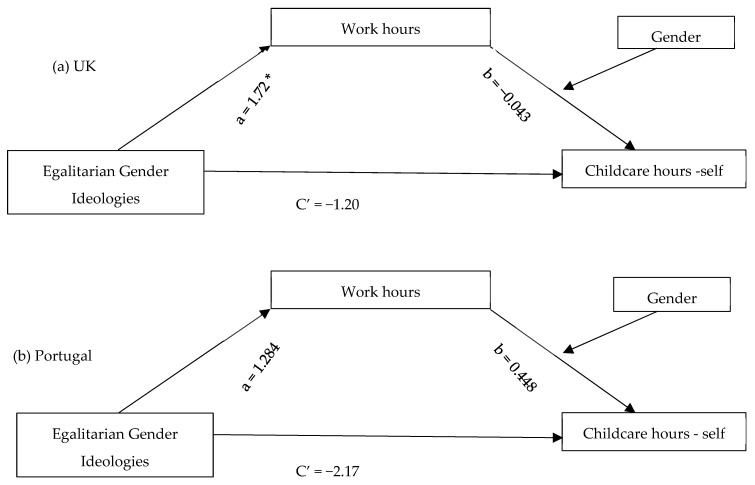
Conditional Indirect Effects of Egalitarian Gender Ideologies on Hours of Childcare Through Work Hours. * *p* < 0.05.

**Figure 5:**
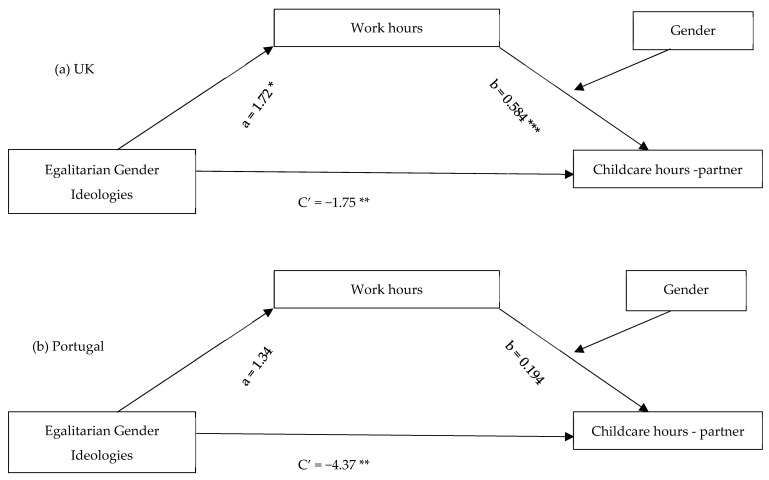
Conditional Indirect Effects of Egalitarian Gender Ideologies on Partner’s Hours of Childcare Through Work Hours. * *p* < 0.05; ** *p* < 0.01; *** *p* < 0.001.

To explore the mediated effect of gender ideologies on childcare involvement through work hours and its moderation by gender in both countries, model 14 of the PROCESS program (Hayes, 2022), developed by Preacher and Hayes (Hayes, 2013; Preacher et al., 2007), was applied. All analyses used bias-corrected bootstrap estimates and 95% confidence intervals of the indirect (mediated) effects and the overall moderated mediation model (Figure 3, Figure 4 and Figure 5). 

Considering direct effects, egalitarian gender ideologies had a positive relationship with working hours for parents in the UK but not in Portugal (Figure 3, Figure 4 and Figure 5(path a)). The same was true for the relationship between work hours and involvement in childcare tasks and partner’s share of childcare hours, which were only significant for parents in the UK.

For parents in both countries, gender ideologies had a direct negative effect on their partner’s hours of childcare (Figure 5(path C’)).

As demonstrated in Table 2, work hours were a significant mediator in all three models, reflected in significant conditional indirect effects of gender ideologies on involvement in childcare tasks for mothers in the UK, and on hours of childcare for both parents in the UK, but not for parents in Portugal. Gender moderated the mediation effect of work hours for parents in the UK, as the index of moderated mediation was negative with bootstrap confidence intervals entirely below zero for partners’ hours of care [−0.689, −0.042]. The indirect effect of gender ideologies on partners’ hours of care, examined through work hours, was stronger for men than for women in the UK. This meant that the more parents believed in gender equality, the more their partners were likely to spend time on childcare, particularly among men.

## 4. Discussion

The current study compared how gender ideologies differ within two national contexts and how they relate to caregiving behaviour, contributing to the understanding of the social psychological mechanisms underlying persistent gender inequalities in family life. The findings revealed notable cross-national differences in parental involvement in paid labour and childcare division and gender ideologies between parents in the UK and Portugal. The results contribute to the understanding of the cross-national differences in the mechanisms linking gender ideologies to childcare practices and demonstrate the gendered nature of work-family dynamics.

Parents in the UK reported significantly more weekly hours dedicated to childcare compared to their Portuguese counterparts. This is consistent with data from Portugal, which indicates that the country is among the European countries where children are least likely to be cared for solely by their parents (Eurostat, 2025). Notably, UK mothers reported the highest number of childcare hours, surpassing both UK fathers and Portuguese mothers.

Regarding paid work, Portuguese parents worked longer weekly hours than UK parents. This difference was particularly salient for mothers, with Portuguese mothers working significantly more than UK mothers. Such results confirm that labour market participation for mothers in Portugal is less constrained by childcare barriers when compared to mothers in the UK (Eurofound, 2024). Additionally, no significant gender differences in paid working hours were observed among Portuguese parents. These patterns reveal an interaction between labour market participation, childcare expectations, and structural support: in Portugal, universal childcare access and gender-neutral parental leave facilitate maternal labour force participation, whereas in the UK, childcare demands constrain mothers’ paid work. Taken together, the results echo previous research that shows parents’ paid work hours strongly influence childcare involvement; more hours in paid work mean less time for caregiving (e.g., Bünning, 2020; Gaunt & Deutsch, 2024; Kaplan & Offer, 2022).

Across both countries, mothers undertook significantly more housework than fathers, aligning with previous research (e.g., Altintas & Sullivan, 2016; Pinho et al., 2025). However, UK fathers reported significantly more housework involvement than Portuguese fathers, while no significant differences were found in the housework contributions of mothers between the two countries. These results are in line with previous research that demonstrated that within households in Portugal, couples have less inclination to share or perform housework themselves, often leading to more outsourcing of chores when compared to UK households (Van der Lippe et al., 2018).

When examining involvement in childcare tasks, mothers in both contexts reported greater involvement in physical, emotional, and responsibility-related aspects of care than fathers (e.g., Horne et al., 2018). Furthermore, parents in the UK, irrespective of gender, showed higher levels of involvement across all childcare domains compared to Portuguese parents. British fathers, in particular, were significantly more engaged in physical childcare tasks than Portuguese fathers, contrary to previous research (Monteiro et al., 2017; Norman & Elliot, 2015). British mothers also reported significantly higher involvement in emotional caregiving compared to Portuguese mothers (Labbas & Stanfors, 2023). The finding that British fathers reported significantly greater involvement in physical childcare tasks than Portuguese fathers can be interpreted through Finch’s (2007) concept of *displaying families*. In the UK, contemporary ideals of “good fatherhood” increasingly emphasise visible, hands-on caregiving, particularly in routine physical tasks such as feeding, bathing, and bedtime. By actively engaging in these practices, British fathers are not only *doing* childcare but also *displaying* family in ways that align with cultural expectations of egalitarian parenting. Such displays serve to signal (to partners, peers, and wider social audiences) that they are modern, involved fathers, thereby securing social recognition of their role. However, a critical reading suggests that these practices may function more as symbolic performances than as evidence of fully equal parental responsibility. Finch’s framework draws attention to how displays must be legible to relevant audiences. For British fathers, participation in visible and easily recognisable childcare tasks may secure legitimacy without necessarily challenging the enduring maternal dominance in the less visible domains of emotional and responsibility-related care. Thus, while British fathers’ increased involvement marks a shift in the cultural scripts of fatherhood, it also illustrates the limitations of display as a measure of substantive gender equality in family life.

In terms of gender ideologies, mothers in both countries endorsed more egalitarian views than fathers, confirming previous findings (Langsæther & Knutsen, 2024). Interestingly, Portuguese parents expressed more egalitarian gender ideologies and lower levels of essentialist beliefs than their UK counterparts. Among fathers, those in Portugal held significantly lower essentialist perceptions than those in the UK. This partly contributes to the explanation of why parents in Portugal, irrespective of gender, have higher involvement in paid work and a lower gender gap in childcare. Meaning believing that men and women should be equally involved in both domains (work and family) and are similarly capable of caring for their children might explain the narrower difference between the tasks and time mothers and fathers in Portugal devote to caring for their children when compared to the UK, where mothers are much more involved in childcare and less involved in paid work when compared to fathers.

The relationship between gender ideologies and paid work also varied by country. In the UK, results show that more egalitarian gender ideologies were positively associated with greater working hours, a pattern not observed in Portugal. This finding is particularly surprising among British men, as it would be expected that egalitarian values would be associated with reductions in paid labour. Contrary to previous research, these results show that egalitarian ideologies do not necessarily motivate individuals to reduce their own paid work in pursuit of greater equal-sharing within the household (e.g., Pinho & Gaunt, 2021).

Importantly, gender ideologies were found to negatively influence partner hours of childcare in both countries, suggesting a compensatory dynamic within couples. Nonetheless, work hours emerged as a significant mediator in the UK only. Specifically, only among UK parents, a significant indirect effect was observed, indicating that individuals with more egalitarian ideologies, through their increased paid work hours, had partners who reported doing more childcare, with stronger effects observed among fathers. This surprising outcome suggests a disconnect between egalitarian beliefs and actual family arrangements. Even when individuals support a more equal division of labour, the behaviours associated with these beliefs may inadvertently increase reliance on traditional caregiving patterns, particularly when increased paid work hours by one partner necessitate greater unpaid care by the other. In this case, egalitarianism was not linked to more equal sharing of care but rather to a reallocation of responsibilities that may deepen gender asymmetries in the home.

Country-specific family policies, reconciliation measures, and workplace norms help explain these cross-national differences. In the UK, Shared Parental Leave (SPL) theoretically allows parents to divide leave after childbirth, but uptake remains low, particularly among fathers, due to financial disincentives and workplace cultures that implicitly discourage men from taking leave (e.g., Clifton-Sprigg et al., 2024). Flexible work arrangements are uneven across sectors, making fathers’ ability to balance paid work and childcare contingent on supportive supervisors or organisational norms. By contrast, Portugal provides a non-transferable father-only leave quota with financial compensation, alongside universal childcare and flexible part-time work opportunities, creating stronger institutional incentives for paternal participation. Nonetheless, persistent cultural norms around gendered household roles mean Portuguese fathers may still participate less in daily physical childcare tasks than British fathers, despite high egalitarian ideologies.

Workplace norms further shape caregiving behaviour. UK workplaces that actively support flexible scheduling, remote work, and visible male caregiving role models enable fathers to engage in childcare without professional penalties. Conversely, workplaces emphasising long hours and constant availability reinforce compensatory dynamics, where mothers undertake more childcare even when fathers endorse egalitarian values. In Portugal, policy alignment with normative expectations reduces stigma around paternal leave, but the gendered division of housework persists, indicating that structural supports must be complemented by cultural change to promote genuine equality across all domains of unpaid care.

Overall, these findings highlight the importance of context-specific factors in shaping parental behaviour. The stronger mediation and moderation effects observed in the UK suggest a socio-cultural context in which gender ideologies may be more tightly coupled with structural work-family arrangements. As Portugal has more extensive family policy supports, such as universal public childcare and more gender-neutral parental leave, these may buffer the relationship between individual ideology and the division of family roles. In such contexts, caregiving decisions may be enabled to a higher extent by available institutional support and structural norms. The results further underscore the limits of ideology as a sole driver of change, particularly in environments where structural barriers or normative pressures constrain individuals’ ability to act on their beliefs, echoing previous research that has shown that egalitarian ideologies are also common among couples who have a semi-traditional division of roles (Gaunt et al., 2022).

## 5. Policy Implications

The findings have several important policy implications for promoting gender equality in both paid and family labour. First, the persistent gender disparities in childcare and housework highlight the need for policies that support more balanced parental involvement. In both the UK and Portugal, governments should consider expanding access to affordable, high-quality childcare services and promoting flexible work arrangements for both mothers and fathers. Second, the greater sensitivity of UK fathers’ involvement to working hours and egalitarian gender ideologies suggests that policies aimed at reducing work–family conflict, such as paid parental leave targeted specifically at fathers, may be especially effective in encouraging paternal participation. In the UK, for instance, uptake of *Shared Parental Leave* has remained low, partly due to financial disincentives and workplace stigma (Clifton-Sprigg et al., 2024). Strengthening the policy by introducing a *non-transferable, adequately paid father quota* (as already exists in Portugal) could encourage higher levels of engagement in early childcare. Beyond leave provision, workplace cultures that normalise and support active fatherhood require concrete measures. For example, ensuring that fathers who take parental leave are not penalised in career progression, encouraging employers to adopt “right-to-request” flexible schedules for men as well as for men, and promoting visible senior role models who combine caregiving with professional responsibilities. Third, given the stronger egalitarian ideologies in Portugal but less behavioural translation, there is a need for mechanisms that bridge the gap between attitudes and practices. Portugal’s policy of allocating a mandatory father-only leave quota is progressive, but its effectiveness could be enhanced by complementary interventions such as public awareness campaigns valorising paternal caregiving, employer incentives (e.g., subsidies or tax benefits for firms that support shared caregiving roles), and monitoring schemes to ensure equitable access for fathers. Finally, cross-national differences underscore the importance of tailoring family and labour policies to specific cultural and institutional contexts. Interventions must address both structural barriers and cultural norms to effectively promote gender equity in family and professional spheres.

## 6. Limitations and Future Research

Several limitations of this study should be noted. First, both samples were disproportionately composed of highly educated parents. Prior research suggests that families with higher educational attainment and income levels are generally less likely to endorse traditional gender attitudes (e.g., Gaunt & Deutsch, 2024). As a result, the range and overall levels of gender ideologies, biological essentialism observed in this study may have been comparatively limited. Therefore, incorporating a more diverse sample in future research may reveal a broader range of childcare and paid work practices. Another limitation lies in the exclusive reliance on self-report measures, which may be subject to social desirability bias. Future research should explore how broader cultural, economic, and policy frameworks shape the interplay between gender ideologies, labour division, and parental involvement across diverse family systems.

An additional limitation concerns the different sampling procedures employed, namely sampling within a nationally representative framework versus recruitment through institutional access. These approaches are not fully equivalent, as they capture different population segments and may reflect distinct forms of bias. This difference has implications for the comparability and generalisability of findings, and should be borne in mind when interpreting the results of this study.

Finally, the cross-sectional design of our study prevents causal inference, and all models assume directionality from ideologies to behaviours, which may be reciprocal. Future research could benefit from longitudinal designs and observational measures of childcare.

## 7. Conclusions

These findings underscore the importance of contextualising gender beliefs within broader cultural and institutional systems. This study provides important insights into cross-national and gendered patterns of parental involvement, work hours, and gender ideologies in the UK and Portugal. The results highlight substantial differences between the two countries, with UK parents, particularly mothers, reporting greater involvement in childcare and housework and fewer paid working hours when compared to Portugal. In both contexts, mothers consistently bear the greater share of unpaid labour. Notably, UK fathers demonstrated higher engagement in childcare and housework than Portuguese fathers; however, the gender gap between parental childcare involvement in Portugal was smaller. The stronger mediation and moderation effects found among UK fathers indicate that their participation is more sensitive to variations in work hours and egalitarian gender ideologies than that of mothers. The findings also demonstrate that gender ideologies play a complex role in shaping family labour patterns. In the UK, egalitarian beliefs were associated with both greater work hours and increased involvement in childcare. Still, this relationship was significantly mediated by actual working time and moderated by gender. These patterns were not observed in Portugal, where gender ideologies appeared less predictive of behavioural outcomes. Overall, this study emphasises the need to consider both structural (e.g., work hours and national context) and social psychological (e.g., gender beliefs) characteristics when examining parental roles. It also underscores the importance of gender-sensitive policies that support equitable sharing of paid and unpaid work, especially in contexts where traditional divisions remain entrenched.

## Figures and Tables

**Table 1:** The Demographic Characteristics of the Participants.

	UK (*n* = 1049)		Portugal (*n* = 155)	
	Mothers (*n* = 492)	Fathers (*n* = 557)	Mothers (*n* = 80)	Fathers (*n* = 75)
Number of children				
1	28.7%	31.3%	40%	41.3%
2	52%	50.6%	51.2%	42.7%
3–5	18.1%	17.5%	8.8%	16%
6–9	1.2%	0.6%	0%	0%
Age				
18–24	0.2%	0.4%	0%	1.4%
25–34	25.8%	16.6%	26.3%	16.2%
35–44	68.7%	63.2%	69.8%	61.8%
45–54	4.8%	16.8%	3.9%	20.6%
55+	0.4%	3.1%	0%	0%
Education				
Less than high school	5.5%	8.1%	5.1%	20%
High school diploma	21.4%	18.1%	17.9%	18.7%
Some college education	2.3%	0.79%	7.7%	6.7%
Academic degree	70.8%	73%	69.3%	54.6%
Monthly personal income				
≤GBP 590	15.4%	3.6%	2.6%	4.1%
GBP 591–GBP 1450	34.5%	11.3%	71.4%	53.4%
GBP 1451–GBP 2600	32.6%	43.8%	11.7%	11%
GBP 2601–GBP 3740	11.3%	24.6%	6.5%	17.8%
≥GBP 3741	6.2%	16.7%	7.8%	13.7%

**Table 2:** Bias-Corrected Bootstrap Estimates for Mediation and Moderated Mediation Analyses.

	Mediation by Work Hours								
	Women			Men			Moderated Mediation		
		95% CI			95% CI			95% CI	
	Estimate	Lower	Upper	Estimate	Lower	Upper	Estimate	Lower	Upper
UK									
Childcare tasks	−0.075 ***	−0.133	−0.024	−0.025	−0.056	0.004	−0.050	−0.115	0.003
Childcare hours—self	−0.752 ***	−1.56	−0.098	−0.413 ***	−0.084	−0.059	−0.339	−0.976	0.004
Childcare hours—partner	0.313 ***	0.182	0.732	0.660 ***	0.093	1.26	−0.347 ***	−0.689	−0.042
Portugal									
Childcare tasks	−0.006	−0.059	0.019	0.002	−0.014	0.021	−0.008	−0.067	0.021
Childcare hours—self	−0.540	−2.74	0.468	0.018	−0.862	0.601	−0.558	−2.82	0.758
Childcare hours—partner	−0.299	−2.28	0.382	−0.019	−0.984	0.488	−0.279	−2.16	0.798

Note: *** p < 0.001.

## Data Availability

The raw data supporting the conclusions of this article will be made available by the authors upon reasonable request.
